# Crucial Role of *Foxp_3_* Gene Expression and Mutation in Systemic Lupus Erythematosus, Inferred from Computational and Experimental Approaches

**DOI:** 10.3390/diagnostics13223442

**Published:** 2023-11-14

**Authors:** Zahra Birjan, Khalil Khashei Varnamkhasti, Sara Parhoudeh, Leila Naeimi, Sirous Naeimi

**Affiliations:** 1 Department of Genetics, College of Science, Kazerun Branch, Islamic Azad University, Kazerun 73, Iran; 2 Department of Medical Laboratory Sciences, Faculty of Medicine, Kazerun Branch, Islamic Azad University, Kazerun 73, Iran

**Keywords:** systemic lupus erythematosus, polymorphism, *Foxp_3_*, *rs2280883*, *rs3761549*, machine learning, deep learning

## Abstract

The impaired suppressive function of regulatory T cells is well-understood in systemic lupus erythematosus. This is likely due to changes in *Foxp_3_* expression that are crucial for regulatory T-cell stability and function. There are a few reports on the correlation between the *Foxp_3_* altered expression level and single-nucleotide polymorphisms within the *Foxp_3_* locus. Moreover, some studies showed the importance of *Foxp_3_* expression in the same diseases. Therefore, to explore the possible effects of single-nucleotide polymorphisms, here, we evaluated the association of IVS9+459/rs2280883 (T>C) and −2383/rs3761549 (C>T) *Foxp_3_* polymorphisms with systemic lupus erythematosus. Moreover, through machine-learning and deep-learning methods, we assessed the connection of the expression level of the gene with the disease. Single-nucleotide polymorphisms of *Foxp_3_* (IVS9+459/rs2280883 (T>C) and −2383/rs3761549 (C>T)) were, respectively, genotyped using allele-specific PCR and direct sequencing and polymerase chain reaction-restriction fragment length polymorphism, in 199 systemic lupus erythematosus patients and 206 healthy age- and sex-matched controls. The Statistical Package for the Social Sciences version 19 and Fisher’s exact and chi-square tests were used to analyze the data. Moreover, six machine-learning models and two sequential deep-learning models were designed to classify patients from normal people in the E-MTAB-11191 dataset through the expression level of *Foxp_3_* and its correlated genes. The allele and genotype frequencies of both polymorphisms in question were found to be significantly associated with an increased risk of systemic lupus erythematosus. Furthermore, both of the two single-nucleotide polymorphisms were associated with some systemic-lupus-erythematosus-related risk factors. Three SVM models and the logistic regression model showed an 81% accuracy in classification problems. In addition, the first deep-learning model showed an 83% and 89% accuracy for the training and validation data, respectively, while the second model had an 85% and 79% accuracy for the training and validation datasets. In this study, we are prompted to represent the predisposing loci for systemic lupus erythematosus pathogenesis and strived to provide evidence-based support to the application of machine learning for the identification of systemic lupus erythematosus. It is predicted that the recruiting of machine-learning algorithms with the simultaneous measurement of the applied single nucleotide polymorphisms will increased the diagnostic accuracy of systemic lupus erythematosus, which will be very helpful in providing sufficient predictive value about individual subjects with systemic lupus erythematosus.

## 1. Introduction

To avoid responsiveness against self-antigens and reduce autoimmunity risk, the immune system has evolved immunological tolerance mechanisms which are categorized as central and peripheral tolerance. The primary deletion of autoreactive T or B cells take place by central tolerance, within the primary (thymus and bone marrow) lymphoid organs. Nevertheless, central tolerance is imperfect and self-reactive cells continuously escape into the periphery. Peripheral tolerance is the inactivation key of autoantigen-recognizing T or B cells which appear in the periphery [1,2]. A unique subset of CD_4_^+^ T cells, known as regulatory T (Treg) cells, are essential mediators of peripheral tolerance to self-antigens. These specialized lymphocytes, with regulatory functions in restraining immune responses [3,4,5], arise during thymic-derived T-cell maturation and are characterized by the expression of the interleukin-2 receptor alpha (IL-2Rα) chain (CD_25_), and the forkhead box P_3_ (Foxp_3_) transcription factor [6]. The master forkhead/winged-helix transcription factor of Foxp_3_ controls the regulation, differentiation, and suppressor function of Treg cells. In contrast, function-impaired Treg cells develop systemic autoimmune diseases which have been found to be associated with mutations on the *Foxp_3_* gene (located on the X chromosome in the Xp11.23 position) [7,8,9,10]. A common one includes systemic lupus erythematosus (SLE) with prevalence rates varying between 3.7/100,000 person-years [11]. SLE is a multisystem, complex, autoimmune disease involving progressive organ damage with the direct contribution of auto-antibodies and self-reactive T cells to its pathologic changes [12,13]. Impaired immune system function in SLE has recently been reported to be associated with single nucleotide polymorphisms (SNPs) in the *Foxp_3_* gene which can alter its expression level and impair the suppressive function of Tregs [3]. Two promoter (−2383/rs3761549 (C>T)) and intronic (IVS9+459/rs2280883 (T>C)) polymorphisms of *Foxp_3_* have been reported to be associated with autoimmune disease risk [14].

Furthermore, gene expression analysis advances our understanding about the underlying molecular mechanisms of SLE. Nowadays, machine learning is a developing area that is known as a revolution in science. Machine learning and its more developed field called deep learning could be represent a solution for the big data interpretation challenge, and used to obtain understandable knowledge from massive gene expression data and facilitate the ability to predict changes in SLE disease [15]. There are various machine-learning methods designed to solve classification problems. One of them is called logistic regression, which uses a sigmoid unit to classify each piece of data based on some inputs as features. The performance of a logistic regression is evaluated by a parameter called loss. A more extended logistic regression with various units and several layers is called a neural network, which is the basic unit of a deep-learning model [16]. Therefore, a logistic regression model is also known as the simplest deep-learning model. For the time being, neural networks are suggested as the best way to solve big data classification problems, while machine-learning models are better for datasets with a small size. Recently, some studies have used these models to perform classifications based on gene expression data in biological challenges [17].

The core finding from the present functional study may fill the existing gaps in our understanding about genetic factors predisposing to SLE and provide a promising way to utilize genetic computational methods for the prediction of risk for SLE.

To shed new light on the molecular mechanism underlying the development of SLE, the present study aimed to realize the probable association of IVS9+459/rs2280883 (T>C) and −2383/rs3761549 (C>T) *Foxp_3_* polymorphisms and also the association of the expression of *Foxp_3_* with SLE through in vitro and machine-learning methods.

## 2. Methods

### 2.1. Experimental Design

For our study, 199 SLE blood samples were collected from patients whose disease had been diagnosed by rheumatologist (based on the proper constellation of clinical (butterfly rash, oral ulcers, single urine: protein/creatinine ratio or 24 h urine protein, >0.5 g, seizures, psychosis, myelitis, and leukopenia) findings and immunological evidence (including ANA level, anti-dsDNA antibodies, and low complement) at Hafez Hospital Lupus Clinic (Shiraz, Iran). Blood samples were also collected from 206 age- and sex-matched healthy subjects (controls) from the organization of the blood transfusion (Shiraz, Iran). All samples were kept in the Autoimmune Diseases Research Center of Shiraz University of Medical Sciences (Shiraz, Iran) until experimental analysis. Subjects with other co-occurrent autoimmune and underlying diseases were excluded. The study protocol was approved by the Ethics Committee of the Islamic Azad University—Kazerun Branch (IR.IAU.KAU.REC.1398.044) and written informed consent was provided to gain consent of research participation.

### 2.2. DNA Isolation and Quality Control

Genomic DNA was extracted from a total blood sample volume of 200 µL using the DNP™ DNA Extraction kit (DNP Extraction Kit, Sinagen Company, Tehran, Iran) and was stored frozen at −20 °C for later use. NanoDrop ND-2000 (Thermo, Wilmington, NC, USA) was used for DNA concentration and quality assessment.

### 2.3. Genotyping

Selected polymorphic sites (IVS9+459/rs2280883 (T>C) and −2383/rs3761549 (C>T)) were genotyped by two independent PCR methods.

The −2383/rs3761549 (C>T) polymorphism was amplified by restriction fragment length polymorphism (RFLP) technique. Amplification program, primers, restriction enzyme, and product sizes are shown in Table 1. A total of 10 µL of PCR product was added to 0.5 µL *BseNI* (*BsrI*) restriction enzyme, 2.5 µL buffer, and 18µL nuclease-free water. The mixture then incubated for 4 h at 65 °C. Next, 15 µL of each digested PCR product containing a 3 µL loading buffer was loaded into a lane of the 3% agarose gel. The DNA bands were then visualized on the UV transilluminator and images were taken with a gel documentation system (UVITEC, UK). Finally, the genotypes of −2383/rs3761549 (C>T) SNP were determined.

IVS9+459/rs2280883 (T>C) was genotyped through allele-specific PCR (AS-PCR) (amplification program, primers, and product sizes are shown in Table 1) and direct sequencing method. Direct sequencing of PCR products recovered by the GEL/PCR Purification Kit (Favorgen Biotech Corp., Ping-Tung, Taiwan) was performed using Genetic Analyzer 3130 x (Applied Biosystems, Waltham, MA, USA). Sequences were analyzed with the CodonCode Aligner V.5.1.5 software (CodonCode Corporation, Centerville, MA, USA).

### 2.4. Data Collection and Preprocessing

We searched for microarray expression datasets in the ArrayExpress (https://www.ebi.ac.uk/arrayexpress/) and GEO (https://www.ncbi.nlm.nih.gov/geo) databases on 7 July 2022, in the current study. Various datasets were selected as our first-level candidates; among them, we chose the E-MTAB-11191 dataset from the ArrayExpress database. The selection criteria were based on the number of samples, study design, and the platform in use. The platform in use in the current study was Affymetrix Human Genome U133 Plus 2.0 Array. We first downloaded raw CEL files and then generated the expression matrix through the RMA method in the affy package in the R environment. The package is developed to generate and modify expression matrices from the Affymetrix platform series. The data values were then normalized and scaled into log2 + 1 format. The matrix was then annotated with Ensembl IDs, Gene Symbols, and Entrez IDs. We did not remove any genes through typical methods such as CPM (counts per million) because we had a specific target gene to study.

### 2.5. Machine-Learning Model Design

First of all, we extracted the expression level of the *Foxp_3_* gene from the expression matrix. The gene had 3 probe IDs; therefore, we considered all of them in our model. The data was first transformed in a way that columns were considered our features (genes), and rows were our labels (patients and normal). We had 101 samples; among them, 17 were normal, and 84 were patients. The data were first scaled between zero and one (a common method in machine-learning models) by the following formula:

Gi−min(G))/(max(G)−min(G)

in which *G* represents the expression value of the gene in the patients *i*, and *min* and *max* values of *G* are the minimum and maximum values of the gene among all patients.

Six machine-learning models were created to classify them based on the expression level of the *Foxp_3_* gene, including linear regression (LR), support vector machine (SVM) with RBF (SVM_1), linear (SVM_2), and poly (SVM_3) kernels, decision tree (DT), and extra-tree classifier (ETC). To train the models, we shuffled and divided 70% of the data into the training datasets and 30% into the testing datasets. For that purpose, we used the train_test_split function from the sklearn library in Python. Each model was first trained on the training dataset and then evaluated on the test datasets.

### 2.6. Co-Expression Network

In order to find genes associated to *Foxp_3_*, the co-expression network analysis was performed. The expression matrix of *Foxp_3_* was extracted. Afterwards, the Pearson correlation test was executed between the gene and all other genes in the main expression matrix. Those genes with a correlation coefficient (CC) > 0.8 and CC < −0.8 were selected.

### 2.7. Deep-Learning Model

Two deep-learning models were designed. We used both the Keras and the TensorFlow libraries in the Python environment. Keras is a branched library from TensorFlow that is developed for deep-learning usages. It generally supports two types of models, including sequential and functional models. In the current study, both models were sequential models. In the first model, we only considered *Foxp_3_* probe IDs as our features. The model had 2 hidden layers with 10 and 5 units, respectively. Moreover, there was another output layer with one unit. The activation function for hidden layers was carried out then, and the output layer was sigmoid. Bias for all layers was considered zero at the first epoch, and the weights were random numbers. Adam was considered as the model optimizer, and, because it was a binary classification, we considered binary cross-entropy to calculate our loss. Moreover, the learning rate was set at a 0.0001 value. The model was trained with 2000 epochs, and the validation dataset was considered 0.25 of the total number of train samples. In addition, at the end of the training, the model was evaluated by the test dataset.

For the second model, we considered all genes with a correlation coefficient > 0.8 or <−0.8 with *Foxp_3_*. We had three hidden layers in the second model with 25, 25, and 12 units, respectively. Other parameters were similar to the first model. However, in this model, because of the large number of features, we did not consider the test dataset, and only the validation dataset with 33% of total samples was considered. We utilized this method because, if the number of samples became less than the number of features, the model could not classify very well.

### 2.8. Software and Statistics

All statistical analysis for the genotyping part was performed in SPSS Statistics 19 software. The significance differences in genotype and allelic frequencies between two groups were verified by the Hardy–Weinberg (HW) equilibrium and chi-square test. Bonferroni corrections were applied to correct for multiple comparisons, and the threshold for statistical significance was set at ≤0.05. In the machine-learning part, all mathematical and statistical calculations were performed in R and Python environments. We applied R version 4.0.1 and R Studio for data preparation and basic statistical tests. We applied Python for deep learning, machine learning, and model evaluation in the Google Colab (https://colab.research.google.com/) environment on 15 July 2022. The runtime was set on TPU, which is developed for better execution of machine-learning projects. Moreover, figures were depicted using Python and the matplotlib library.

## 3. Results

The basic demographic data of all the SLE patients are summarized in Table 2.

The results showed that the statistical power in our study were: (1) the associations between both *Foxp_3_* (IVS9+459/rs2280883 (T>C) and −2383/rs3761549 (C>T)) gene polymorphisms and SLE risk, and (2) the 81% accuracy of the three SVM models and the logistic regression model when performing classifications based on gene expression data in biological challenges about genetic factors predisposing to SLE.

**Figure 1:**
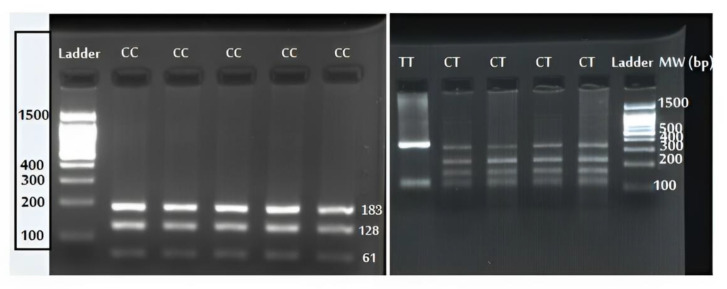
Agarose gel electrophoresis of rs3761549 polymorphism and its restricted fragments obtained by BseNI (BsrI) digestion.

The −2383/rs3761549 (C>T) genotype distribution was in accordance with the Hardy–Weinberg equilibrium (control group, X^2^ = 3.2, df = 2, HWE *p*-value = 0.201; and patient group, X^2^ = 4.7, df = 2, HWE *p*-value = 0.095). The CC, CT, and TT genotype of the −2383/rs3761549 (C>T) polymorphism is shown in Figure 1. As the genotypic and allelic distribution of *Foxp_3_* rs3761549 SNP is summarized in Table 3, the CT- and TT-genotype frequencies were significantly higher in the SLE patients than controls. Moreover, our results indicate that the T allele of rs3761549 is a risk allele for SLE development (Table 3). Regarding the association of rs3761549 (C>T) polymorphism with SLE risk factors such as antinuclear antibody (ANA), anti-double-stranded DNA (anti-dsDNA), complement (C3/C4), and white blood cell count (WBC), only a significant relationship was found between CT-genotype carriers and anti-dsDNA (Table 4).

**Figure 2:**
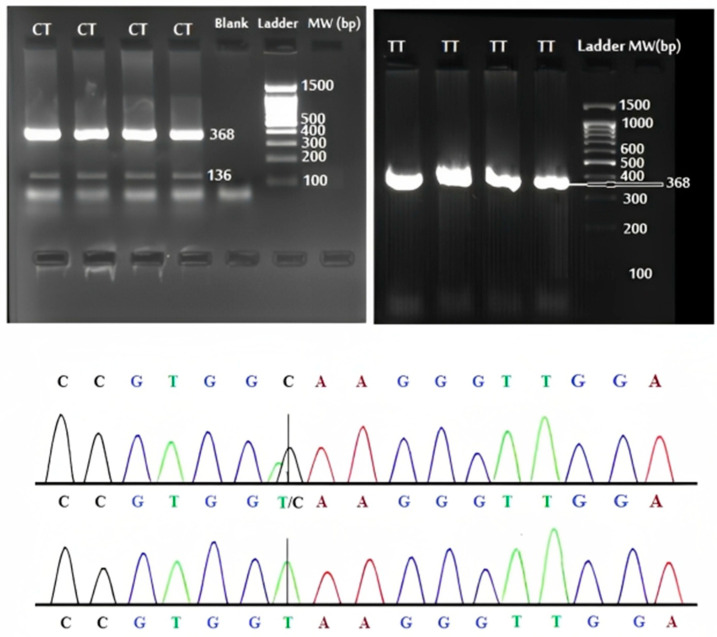
The electrophoretic and sequencing results of PCR products of rs2280883 polymorphism.

An evaluation of the Hardy–Weinberg equilibrium for the rs2280883 polymorphic loci showed a nonsignificant deviation in both the control and patient population (control group, X^2^ = 1.4, df = 2, HWE *p*-value = 0.496; and patient group, X^2^ = 1.6, df = 2, HWE *p*-value = 0.449). Figure 2 demonstrated the CT and TT genotype confirmed by direct PCR sequencing. The relationships between the rs2280883 risk genotypes and alleles, and susceptibility to SLE were analyzed (Table 3). We found a significantly increased risk for SLE associated with the rs2280883 polymorphism CT genotype and C allele. Except for C3, no statistically significant difference was observed between rs2280883 SNP and various SLE risk factors (Table 4). After Bonferroni correction, both SNPs remained significant.

### 3.1. Foxp_3_ Expression Level Might Efficiently Classify People with or without Lupus Erythematosus through Machine-Learning Methods

We applied six machine-learning models to classify normal people from patients. The results are shown in Table 5. Overall, it is evident that all SVM models and logistic regression could indicate similar outcomes with an accuracy of 81%. On the other hand, decision tree and ETC models were 68% and 74% accurate in the classification problem. All the classification models were designed based on only one gene as our feature. None of the models showed a macro average F1-score of more than 45%. However, the weighted average F1-score of all models was in a similar range between 65% and 72%. The four models listed at first showed the highest metrics based on all methods.

### 3.2. A Deep-Learning Model with More Features: Similar Results to the Model with One Feature

**Figure 3:**
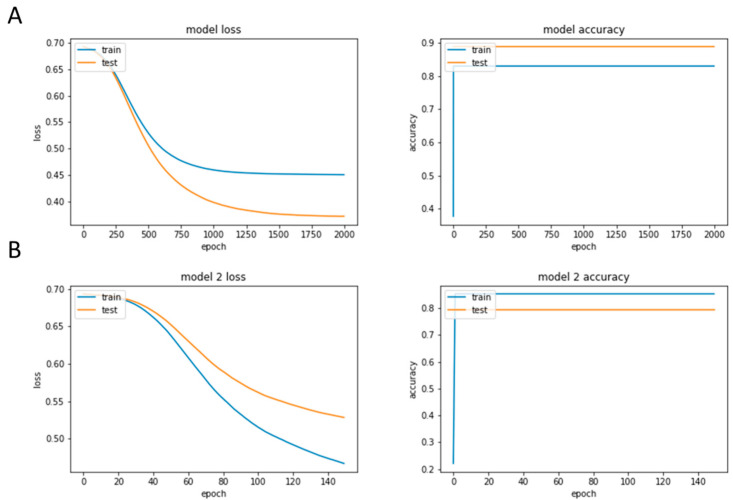
The training history of both models. (**A**) First model with only one feature and (**B**) Second model with correlated genes.

We designed two deep-learning models to assess the performance of neural networks in the classification with such a number of samples. The training history of both models is shown in Figure 3A,B. As the first model had only one feature (*Foxp_3_*), we only considered two layers but a more extensive training time for that (2000 epochs). The model showed an 83% and 89% accuracy for the training and validation datasets, respectively. The loss for both datasets was at the minimum level of its function, and the training was precisely stopped at this point. On the other hand, for the second model, we considered 76 genes that had the highest CC with *Foxp_3_* (Supplementary Table S1). The expression level of all genes was normalized and scaled between zero and one then. We designed the model with three hidden layers, as the number of features was larger. However, we reduced the number of epochs to achieve better results. The model showed an 85% accuracy for the training set, which is larger than the previous model. However, the validation accuracy could only reach 79%. Therefore, the first model with *Foxp_3_* as its only feature revealed better results compared to the second model with correlated genes. This fact reveals that, despite the low number of samples available for this deep-learning modelling, *Foxp_3_* could classify patients and normal people very well.

## 4. Discussion

It has long been suggested that genetic factors not only enhance the risk for the development of SLE, but also are able to play important roles in the pathogenesis of this disease. However, the exact cause of SLE remains elusive [18] and further experiments are still needed. Thus, in this study, we aimed to prove whether the *Foxp_3_* expression and mutation are crucial in lupus erythematosus, through computational modelling and experimental approaches. Despite the success of SNP analyses in the context of assessing the association between genetic determinants and complex diseases, disease-risk SNPs are usually neglected. On the other hand, although the SNP discovery holds great promise, SNPs may not be the single mediator for the relation between genetics and disease. Gene expression information can also increase the power of detecting the overall effect of genetics on disease risk [19]. In this paper, we combined the information of SNPs and gene expression to introduce *Foxp_3_* as a mediator which highlights the clinical significance of our findings.

In the present study, the role of *Foxp_3_* polymorphisms has attracted attention in the SLE pathogenesis. Our main findings suggest that the T allele of *Foxp_3_*−2383 C>T (rs3761549) could be a risk allele and the CT and TT genotypes were associated with developing SLE. Other findings are in agreement with our result; for example, in a Brazilian population, the rs2232368 polymorphism T allele was found to have an association with endometriosis-related infertility [20]. The association between the “CT” genotype of -2383 C/T (rs3761549) polymorphism and Hashimoto’s thyroiditis and Graves’ disease is also reported in the South Indian population [21]. Moreover, we found a significant relationship between CT-genotype carriers and the positive anti-dsDNA antibody. It suggests that the rs3761549 could be considered as a genetic risk factor for SLE susceptibility. Likewise, observing the association of the CT genotype and C allele of IVS9+459 T>C (rs2280883) polymorphism with an increased risk for SLE in our study implies the effect of rs2280883 SNP’s predisposition to SLE. There is also a report indicating the associations between the rs2280883 SNP and psoriasis in a Han Chinese population [22]. The rs2280883 SNP is also linked to an increased risk of Graves’ disease (Tan et al., 2021). Additionally, the association of the TC genotype of *Foxp_3_* rs2280883 was found with the risk of connective-tissue-disease-associated ILD [23]. Findings in other studies, together with the *Foxp_3_*-polymorphism-associated genetic effect on the risk of SLE identified in this study, confirm our hypothesis that *Foxp_3_* polymorphisms might be a proper candidate for use in autoimmune disease screening, including for SLE. However, no evidence for the *Foxp_3_* gene polymorphism association with Graves’ disease and autoimmune Addison’s disease has been found in the UK population [24]. It is clearly the case that there is inconsistency between different ethnic populations concerning the polymorphisms responsible for disease susceptibility. We suggest that the association between such identified true susceptibility loci and SLE and other autoimmune diseases should be evaluated in every population.

On the other hand, in the current study, the potential of the *Foxp_3_* gene expression level in patient classification was evaluated. Our present result is similar to the findings of a previous study which indicate machine-learning algorithms could potentially be applied to identify the gene expression features and subjects with higher degrees of disease activity [25]. Moreover, there are reports about the importance of this gene expression activity in SLE in the Egyptian population [26]. The dataset in use in this study was selected among more than 20 candidate datasets. Unfortunately, we could not select any microarray dataset with a larger number of samples because of the type of study and the platform in use. Therefore, we only considered 101 samples: 17 were without the disease and the others were patients. This means that around 17% of our samples were grouped as control, which is acceptable for a small machine-learning project. However, with the small number of samples, the outcomes showed that the *Foxp_3_* expression levels might classify both groups considerably. A comparison among the deep-learning and machine-learning models shows that the first deep-learning model had the best performance with about 90% accuracy. In this model, we only considered *Foxp_3_* as our feature. We believe that the better performance of the model is due to the number of samples, as the rules of deep learning say the number of features should be much less than the sample number to obtain the best result. On the other hand, among machine-learning models, logistic regression and all support vector machine models showed the same result with an accuracy of 81%. Here, again, we think that the same results are because of the number of control samples. Definitely, if we increase this number, the results would be changed. Therefore, we cannot decide which one of the machine-learning models could classify better. Considering a larger sample size to evaluate the association between these polymorphisms in question with SLE in future studies will resolve the present study’s limitation. Likewise, further studies on patients in a variety of ethnic populations are still required to increase our knowledge base for this gene. It is advantageous that other genetic association studies evaluate other potential mediators, such as DNA methylation.

## 5. Conclusions

To date, although the role of *Foxp_3_* in autoimmunopathies have attracted interest in numerous genetic studies, the present study has been attempted to detect *Foxp_3_* gene expression features and its related polymorphisms as more plausible genetic risk factors for SLE. The present data provide an approach to considering the *Foxp_3_* gene as a strong genetic component with high clinical significance for SLE which could potentially be used to identify the subjects with higher disease susceptibility.

## Figures and Tables

**Table 1:** Amplification program, primers, restriction enzyme, and product sizes used for genotyping of rs3761549 SNP, and amplification program and primers used for genotyping of rs2280883 SNP.

	rs3761549 (Promoter Region)
Type of polymorphism	Single-base C>T
Site of polymorphism	−2383
PCR primers	
Forward:	5′-CTGAGACTTTGGGACCGTAGAC-3′
Reverse:	5′-ACACCACGGAGGAAGAGAAGAG-3′
PCR conditions	
Denaturation:	94 °C, 5 min
Annealing:	64 °C, 30 s
Extension:	72 °C, 7 min
No. of cycles:	35
Restriction enzyme:	*BseNI (BsrI)*
Restriction Enzymes Product Size (bp):	CC (183, 128, and 61 bp) CT (311, 183, 128, and 61 bp) TT (311 and 61 bp)
	rs2280883 (Intronic region)
Type of polymorphism	Single-base T>C
Site of polymorphism	IVS9+459
PCR primers	
T Allele	
Forward:	5′-ACCACCATCCAGGCCAGAG-3′
Reverse:	5′-GTGTGGCGCTAGGATGAAGG-3′
C Allele	
Forward:	5′-AATACACCCCCAACTGGGCA-3′
Reverse:	5′-GTGTGGCGCTAGGATGAAGG-3′
PCR conditions	
Denaturation:	95 °C, 5 min
Annealing:	58 °C, 1 min
Extension:	72 °C, 3 min
No. of cycles:	30
Product Size (bp):	T (368 bp) C (136 bp)

**Table 2:** Demographic characteristics of participants in two groups.

Variables	Controls	Patients	*p*-Value
	N = (206)	N = (199)	
Age, years	40.46 ± 10.4	34.59 ± 10.9	0.223
Range	19–61	14–71	-
Sex			
Male	15 (7.7%)	16 (8%)	0.134
Female	191 (92.3%)	183 (92%)	

**Table 3:** Genotype and allele frequency distribution of rs3761549 and rs2280883 polymorphisms in SLE patients and controls.

Gene	SNP	Controls (n = 206)	Patients (n = 199)	OR (95% CI)	Uncorrected *p*	Corrected *p*
*Foxp_3_*	rs3761549					
	CC	145 (66.1%)	91 (46.7%)	1	<0.001	<0.003
	CT	61 (33.9%)	107 (52.8%)	2.2 (1.4–3.3)		
	TT	(0)	1 (0.5%)	-		
	C	299 (83.1%)	285 (47.5%)	1		
	T	61 (16.9%)	315 (52.5%)	2.2 (1.4–3.3)	<0.001	<0.007
	rs2280883					
	TT	117 (56.4%)	77 (45.6%)	1	0.037	0.045
	CT	89 (43.6%)	122 (54.4%)	0.6 (0.4–0.9)		
	CC	0 (0)	0 (0)	-		
	T	260 (72%)	289 (66%)	1		
	C	143 (28%)	89 (34%)	0.5 (0.4–0.7)	<0.001	<0.005

Note: Corrected *p*-values were calculated by using Bonferroni’s correction.

**Table 4:** Association of rs3761549 and rs2280883 polymorphisms and the SLE developmental risk factors.

rs3761549	Genotypes (%)		OR (95% CI)	*p*-Value
	CC	CT		
ANA Negative Positive C3 Normal Decrease Increase C4 Normal Decrease Increase Anti-ds DNA No Yes WBC Normal Decrease	28 63 52 25 7 66 10 9 47 44 85 4	29 76 58 31 7 76 14 6 39 66 95 10	1 1.16 (0.6–1.2) 1 1 (0.5–2.1) 0.8 (0.2–2.7) 1 1.2 (0.5–2.9) 0.5 (0.19–1.7) 1 1.8 (1.03–3.1) 1 2.2 (0.6–7.5)	0.6 0.7 0.8 0.6 0.3 0.04 0.17
**rs2280883**	**Genotypes** **(%)**		**OR (95% CI)**	***p*-Value**
	**TT**	**CT**		
ANA Negative Positive C3 Normal Decrease Increase C4 Normal Decrease Increase Anti-ds DNA No Yes WBC Normal Decrease	33 73 69 24 6 83 9 8 50 56 99 7	25 65 43 31 8 61 14 7 38 52 83 7	1 1.17 (0.6–1.3) 1 2 (1.07–3.9) 2 (0.6–6.9) 1 2 (0.8–5.2) 1.19 (0.3–3.4) 1 1.2 (0.6–2.1) 1 0.7 (0.4–3.2)	0.6 0.02 0.18 0.1 0.7 0.4 0.7

**Table 5:** Machine-learning results for FOXP3-based model.

	Logistic Regression	SVM_1	SVM_2	SVM_3	Decision Tree	ETC
Accuracy	0.81	0.81	0.81	0.81	0.68	0.74
Macro Avg	0.45	0.45	0.45	0.45	0.40	0.43
Weighted Avg	0.72	0.72	0.72	0.72	0.65	0.69

## Data Availability

The data that support the findings of this study are available from the corresponding author upon reasonable request.

## Supplementary Materials

The following supporting information can be downloaded at: https://www.mdpi.com/article/10.3390/diagnostics13223442/s1.

## Abbreviations

Treg: Regulatory T cells, Foxp_3_; Forkhead box P_3_, IL-2Rα; Interleukin-2 receptor alpha chain, SLE; Systemic lupus erythematosus, SNPs; Single nucleotide polymorphisms, RFLP; Restriction fragment length polymorphism, AS-PCR; Allele-specific PCR, LR; Linear regression, SVM; Support vector machine, DT; Decision tree, ETC; Extra-tree classifier, HWE; Hardy–Weinberg equilibrium, ANA; Antinuclear antibody, anti-dsDNA; Anti-double-stranded DNA, C3/C4; Complement, WBC; White blood cell count.
